# Clinical Subphenotypes of *Staphylococcus aureus* Bacteremia

**DOI:** 10.1093/cid/ciae338

**Published:** 2024-06-25

**Authors:** Maaike C Swets, Zsuzsa Bakk, Annette C Westgeest, Karla Berry, George Cooper, Wynne Sim, Rui Shian Lee, Tze Yi Gan, William Donlon, Antonia Besu, Emily Heppenstall, Luke Tysall, Simon Dewar, Mark de Boer, Vance G Fowler, David H Dockrell, Guy E Thwaites, Miquel Pujol, Natàlia Pallarès, Cristian Tebé, Jordi Carratalà, Alexander Szubert, Geert H Groeneveld, Clark D Russell

**Affiliations:** Department of Infectious Diseases, Leiden University Medical Center, Leiden University, Leiden, The Netherlands; Roslin Institute, University of Edinburgh, Edinburgh, United Kingdom; Department of Methodology and Statistics, Leiden University, Leiden, The Netherlands; Department of Infectious Diseases, Leiden University Medical Center, Leiden University, Leiden, The Netherlands; Centre for Inflammation Research, Institute for Regeneration and Repair, University of Edinburgh, Edinburgh, United Kingdom; Clinical Infection Research Group, Western General Hospital, Edinburgh, United Kingdom; Centre for Inflammation Research, Institute for Regeneration and Repair, University of Edinburgh, Edinburgh, United Kingdom; Edinburgh Medical School, University of Edinburgh, Edinburgh, United Kingdom; Edinburgh Medical School, University of Edinburgh, Edinburgh, United Kingdom; Edinburgh Medical School, University of Edinburgh, Edinburgh, United Kingdom; Edinburgh Medical School, University of Edinburgh, Edinburgh, United Kingdom; Edinburgh Medical School, University of Edinburgh, Edinburgh, United Kingdom; Centre for Inflammation Research, Institute for Regeneration and Repair, University of Edinburgh, Edinburgh, United Kingdom; Medical Microbiology, Royal Infirmary of Edinburgh, Edinburgh, United Kingdom; Clinical Infection Research Group, Western General Hospital, Edinburgh, United Kingdom; Medical Microbiology, Royal Infirmary of Edinburgh, Edinburgh, United Kingdom; Department of Infectious Diseases, Leiden University Medical Center, Leiden University, Leiden, The Netherlands; Department of Clinical Epidemiology, Leiden University Medical Center, Leiden, The Netherlands; Division of Infectious Diseases and International Health, Department of Medicine, Duke University School of Medicine, Durham, North Carolina, USA; Duke Clinical Research Institute, Durham, North Carolina, USA; Centre for Inflammation Research, Institute for Regeneration and Repair, University of Edinburgh, Edinburgh, United Kingdom; Oxford University Clinical Research Unit, Ho Chi Minh City, Vietnam; Centre for Tropical Medicine and Global Health, Nuffield Department of Medicine, University of Oxford, Oxford, United Kingdom; Department of Infectious Diseases, Bellvitge University Hospital, L´Hospitalet de LLobregat, Barcelona, Spain; Bellvitge Biomedical Research Institute, L’Hospitalet de Llobregat, Barcelona, Spain; Centro de Investigación Biomédica en Red de Enfermedades Infecciosas, Instituto de Salud Carlos III, Madrid, Spain; Biostatistics Support and Research Unit, Germans Trias i Pujol Research Institute and Hospital, Badalona, Spain; Department of Basic Clinical Practice, School of Medicine and Health Sciences, University of Barcelona, Barcelona, Spain; Biostatistics Support and Research Unit, Germans Trias i Pujol Research Institute and Hospital, Badalona, Spain; Department of Infectious Diseases, Bellvitge University Hospital, L´Hospitalet de LLobregat, Barcelona, Spain; Bellvitge Biomedical Research Institute, L’Hospitalet de Llobregat, Barcelona, Spain; Centro de Investigación Biomédica en Red de Enfermedades Infecciosas, Instituto de Salud Carlos III, Madrid, Spain; Department of Clinical Sciences, School of Medicine and Health Sciences, University of Barcelona, Barcelona, Spain; MRC Clinical Trials Unit, University College London, London, United Kingdom; Department of Infectious Diseases, Leiden University Medical Center, Leiden University, Leiden, The Netherlands; Department of Internal Medicine–Acute Internal Medicine, Leiden University Medical Center, Leiden, The Netherlands; Centre for Inflammation Research, Institute for Regeneration and Repair, University of Edinburgh, Edinburgh, United Kingdom; Medical Microbiology, Royal Infirmary of Edinburgh, Edinburgh, United Kingdom

**Keywords:** Staphylococcus aureus, bacteraemia, patient stratification, subphenotypes, adjunctive treatment

## Abstract

**Background:**

*Staphylococcus aureus* bacteremia (SAB) is a clinically heterogeneous disease. The ability to identify subgroups of patients with shared traits (subphenotypes) is an unmet need to allow patient stratification for clinical management and research. We aimed to test the hypothesis that clinically relevant subphenotypes can be reproducibly identified among patients with SAB.

**Methods:**

We studied 3 cohorts of adults with monomicrobial SAB: a UK retrospective observational study (Edinburgh cohort, n = 458), the UK ARREST trial (n = 758), and the Spanish SAFO trial (n = 214). Latent class analysis was used to identify subphenotypes using routinely collected clinical data without considering outcomes. Mortality and microbiologic outcomes were then compared between subphenotypes.

**Results:**

Included patients had predominantly methicillin-susceptible SAB (1366 of 1430, 95.5%). We identified 5 distinct, reproducible clinical subphenotypes: (A) SAB associated with older age and comorbidity, (B) nosocomial intravenous catheter-associated SAB in younger people without comorbidity, (C) community-acquired metastatic SAB, (D) SAB associated with chronic kidney disease, and (E) SAB associated with injection drug use. Survival and microbiologic outcomes differed between the subphenotypes. Mortality was highest in subphenotype A and lowest in subphenotypes B and E. Microbiologic outcomes were worse in subphenotype C. In a secondary analysis of the ARREST trial, adjunctive rifampicin was associated with increased mortality in subphenotype B and improved microbiologic outcomes in subphenotype C.

**Conclusions:**

We have identified reproducible and clinically relevant subphenotypes within SAB and provide proof of principle of differential treatment effects. Through clinical trial enrichment and patient stratification, these subphenotypes could contribute to a personalized medicine approach to SAB.

## Teaser

We studied 1430 patients with *Staphylococcus aureus* bacteremia in 3 separate cohorts and used latent class analysis to identify 5 distinct and reproducible clinical subphenotypes. These subphenotypes were associated with differences in mortality, microbiologic outcomes, and response to adjunctive rifampicin.

*Staphylococcus aureus* bacteremia (SAB) has long been recognized as a difficult-to-treat bacterial disease that requires prolonged antimicrobial treatment [1, 2]. Major complications include the development of metastatic foci of infection (up to 37% [3]), recurrence of bacteremia despite appropriate treatment (up to 10% [4]), and death (15%–30% in-hospital [5, 6]). Globally, *S. aureus* accounts for the most overall deaths due to a bacterial pathogen and, specifically, the most deaths associated with bacteremia [7].

A defining clinical feature of SAB is heterogeneity. This encompasses patient characteristics (eg, age and comorbidity), pathogen characteristics, place of acquisition (community or hospital), source of bacteremia, and extent of infection. Currently, there is no consensus on rationalizing this clinical heterogeneity to achieve patient stratification, and clinical trials mainly consider SAB to be a single syndrome. Strategy trials in SAB, which frequently investigate combination antimicrobial therapy, have so far not succeeded in identifying approaches that improve outcomes compared with standards of care [8–10]. However, because of the clinical heterogeneity intrinsic to SAB, it is possible that we fail to identify subgroups of patients who may differentially benefit (or suffer harm) from specific therapies [11]. In contrast, clinically relevant subphenotypes have been identified in similarly heterogeneous diseases including acute respiratory distress syndrome [12], asthma [13], chronic obstructive pulmonary disease [14], and bronchiectasis [15]. In this context, a subphenotype is considered to be a subgroup of patients with a specific disease who exhibit similar traits, such as clinical features, outcomes, or responses to treatment [16]. We aimed to test the hypothesis that clinically relevant subphenotypes can be reproducibly identified among patients with SAB.

## METHODS

### Patient Cohorts

Patients were included from the following 3 cohorts: a retrospective observational cohort study (Edinburgh cohort, n = 458) [17]; the ARREST (Adjunctive rifampicin to reduce early mortality from Staphylococcus-aureus bacteraemia) multicenter, randomized, double-blind, placebo-controlled trial (n = 758) [10]; and the SAFO (Multicentre, randomized, open-label, phase IV-III study to evaluate the efficacy of cloxacillin plus fosfomycin versus cloxacillin alone in adult patients with methicillin-susceptible Staphylococcus aureus bacteremia) randomized clinical trial (n = 214) [8]. The Edinburgh cohort included consecutive adults (aged ≥18 years) with monomicrobial SAB diagnosed between 20 December 2019 and 23 August 2022 in 3 UK hospitals (Supplementary Figure 1). The ARREST trial recruited adults (aged ≥18 years) with monomicrobial SAB in 29 UK hospitals between 10 December 2012 and 25 October 2016 and randomized participants to receive adjunctive rifampicin (600 mg/day or 900 mg/day) or placebo for up to 14 days in addition to standard antibiotic treatment. Exclusion criteria included evidence of rifampicin nonsusceptible *S. aureus*, contraindications to rifampicin, and whether adjunctive rifampicin was considered mandatory. The SAFO trial recruited adults (aged ≥18 years) with monomicrobial methicillin-susceptible *S. aureus* (MSSA) bacteremia in 19 Spanish university hospitals between 31 May 2019 and 24 February 2022 and randomized participants to receive cloxacillin (2 g 6 times/day) plus fosfomycin (3 g 4 times/day) or cloxacillin alone for the initial 7 days of treatment. Exclusion criteria included Child-Pugh class C liver cirrhosis, moderate to severe heart failure, injection drug use (IDU), methicillin-resistant *S. aureus* (MRSA) bacteremia, penicillin allergy, and acute severe acute respiratory syndrome coronavirus 2 infection. Ethical approvals were obtained from the South East Scotland Research Ethics Committee for the Edinburgh cohort study, the London (Westminster) Research Ethics Committee for the ARREST trial, and the Spanish Medicines and Healthcare Products Regulatory Agency and the Bellvitge University Hospital Ethics Committee for the SAFO trial.

### Variables and Definitions

Comorbidities were defined according to the Charlson comorbidity index. Acquisition of infection was categorized according to the definitions used by Friedman and colleagues [18]. The source of infection was the most likely portal of entry of *S. aureus* into the bloodstream. Metastatic infection was defined as the presence of foci of infection remote from the portal of entry thought to have arisen through hematogenous dissemination. All-cause 84-day mortality was recorded in all cohorts. In the Edinburgh cohort, persistent bacteremia was defined as a further positive blood culture during treatment >96 hours after the index blood culture and recurrent bacteremia was defined as a further positive blood culture with the same *S. aureus spa* type within 90 days of stopping treatment [3]. In the ARREST cohort, microbiologic failure was defined as ongoing signs and symptoms of infection and growth of *S. aureus* from blood or a sterile site for >14 days from randomization. Recurrence was defined as growth of *S. aureus* from a sterile site after >7 days of apparent clinical improvement. These were combined into a composite microbiologic outcome referred to as composite microbiologic failure. In the SAFO trial, persistent bacteremia was documented on day 3 and day 7 after randomization.

### Statistical Analyses

Latent class analysis (LCA) was used to look for homogenous subgroups within the larger heterogeneous cohorts of SAB using indicator variables selected based on availability and potential clinical relevance (consensus opinion of C. D. R., M. S., A. C. W., and G. H. G.) [19]. Baseline patient and microbiologic variables were considered to be class-defining variables that were first determined using data from the Edinburgh cohort; this model was then applied to the ARREST and SAFO cohorts. The classes were formed without any consideration of clinical or microbiological outcomes. We excluded variables with >10% missing data, categorical variables with >50% colinearity, any variable with <10% positivity unless considered of high clinical relevance, and any variable contributing <0.5% to the clustering [20]. Nonnormally distributed values were log-transformed for the LCA. Cases with missing values were handled with full information maximum likelihood (FIML), which is generally the preferred method for dealing with missing data in LCA [20]. With FIML, data are not imputed, but all available information is used for calculation of the likelihood contribution of each respondent to the estimation of the model parameters [20].

Model selection was based on a combination of statistical criteria and clinical knowledge. The statistical criteria used were the Bayesian information criteria (BIC), number of classes, and size of smallest class. The BIC is a statistical measure that provides information on the model fit and is best at identifying the correct number of classes if a combination of continuous and categorical data is used [20]. A decrease in the BIC suggests that the addition of more classes is worth the added model complexity [21]. To avoid a local maximum, in which case it would be difficult to replicate our findings, 16 random starting values were used and 50 iterations for each start value were performed. Those solutions were checked to make sure that the same maximum likelihood solution was found. When setting the seed, a fixed starting point for random number generation was established, which ensures reproducibility across different runs of the analysis. After identification of classes, we estimated the posterior probability of class membership for each of the identified classes for each individual and assigned the individual to the class with the highest probability [20]. Given that LCA is a probabilistic method, there is a certain degree of uncertainty in class assignment, which can lead to classification errors. For example, an individual may have a 0.9 chance of belonging to class one and a 0.1 chance of belonging to class two. This individual is then assigned to class one. We correct for misclassification error using the bias-adjusted 3-step LCA [22]. LCA was done using the Latent GOLD 6.0 statistical software package [23].

Cohort characteristics were compared using contingency tables for categorical variables (*χ*^2^ or Fisher exact test) and Mann-Whitney or Kruskal-Wallis tests for continuous variables (which Shapiro-Wilk tests demonstrated to be not normally distributed). To compare class-defining variables between subphenotypes, *z* scores were calculated ($z=\frac{\left(value\;for\;subphenotype\;-mean\;for\;variable\right)}{standard\;deviation\;for\;variable}$). Additional metadata not included as class-defining variables were compared between patients stratified by predicted subphenotype membership. Unadjusted 1-year survival was compared using a Kaplan-Meier survival curve and log-rank test, performed using the *survminer* [24] and *ggplot2* [25] packages in R (RStudio version 2023.06.1 + 524). Unless otherwise stated, analyses and data visualization were done using GraphPad Prism version 10.0.3 for macOS.

## RESULTS

### Cohort Characteristics

Characteristics of the Edinburgh, ARREST, and SAFO cohorts are compared in Table 1. In comparison with the Edinburgh cohort, patients in ARREST were more likely to have SAB originating from skin or soft tissue infections (SSTIs), and patients in SAFO were more likely to have an intravenous catheter as the source of bacteremia. Consistent with previous comparisons of real-life patient cohorts with trial cohorts in SAB [9], 84-day mortality was lower in the ARREST and SAFO control arms compared with the Edinburgh cohort. Patients in the Edinburgh and ARREST cohorts predominantly had infection with MSSA (441 of 458 and 711 of 758, respectively), and the SAFO trial exclusively recruited people with MSSA bacteremia.

**Table 1.** Characteristics of Included Patients

Characteristic	Edinburgh Cohort (n = 458)	ARREST Cohort (n = 758)	SAFO Cohort (n = 214)	*P* Value
Age, y	68 (52–79)	65 (50–76)	65 (54–75)	.08
Sex, male	292 (53.2)	NA^a^	150 (70.1)	.11
Acquisition				<.0001
Community-acquired	181 (39.5)	485 (64.1)	78 (36.4)	
Healthcare-associated	110 (24.0)	140 (18.5)	52 (24.3)	
Nosocomial	167 (36.5)	132 (17.4)	84 (39.3)	
Comorbidity				
Dementia	44 (9.6)	31 (4.1)	7 (3.3)	.0002
Chronic kidney disease	35 (7.6)	138 (18.3)	18 (8.4)	<.0001
Liver disease^b^	57 (12.4)	56 (7.4)	13 (6.1)	.004
Vascular disease^c^	121 (26.4)	NA	59 (27.6)	.77
Prosthetic cardiac material^d^	46 (10.0)	NA	16 (7.5)	.31
Injection drug use	41 (9.0)	83 (11.1)	0	<.0001
Vital signs				
Heart rate, beats per minute	98 (85–110)	94 (82–107)	NA	.005
Temperature, °C	38.2 (37.6–38.8)	37.0 (37.0–38.0)	37.3 (36.5–38.3)	<.0001
Laboratory measurements				
Hemoglobin, g/L	114 (100–129)	107 (93–122)	NA	<.0001
Creatinine, µmol/L	90 (65–138)	80 (61–131)	80 (62–123)	.05
C-reactive protein, mg/L	156 (69–273)	150 (87–218)	NA	.15
*Staphylococcus aureus* bacteremia characteristics				
Methicillin-resistant *S. aureus*	17 (3.7)	47 (6.2)	0	<.0001
Infective endocarditis	35 (7.6)	40 (5.3)	15 (7.0)	.21
Other metastatic foci^e^	99 (21.6)	203 (26.8)	49 (22.9)	.11
Source of bacteremia				<.0001
Unknown	163 (35.6)	221 (29.2)	70 (32.7)	
Intravenous catheter	93 (20.3)	141 (18.6)	68 (31.8)	
Skin or soft tissue infection	88 (19.2)	293 (38.7)	39 (18.2)	
Other	61 (13.3)^f^	55 (7.3)^g^	20 (9.3)	
Respiratory	28 (6.1)	29 (3.8)	4 (1.9)	
Urine	25 (5.5)	19 (2.5)	13 (6.1)	
All-cause 84-day mortality	121 (26.4)	56/388 (14.4)^h^	17/110 (15.5)^h^	<.0001

Continuous values are shown as median (interquartile range). Categorical variables are shown as count (%). Variables not available in the ARREST dataset are represented as NA. Vital signs and laboratory measurements were recorded at the time of the index blood culture in the Edinburgh and SAFO cohorts. In the ARREST trial, baseline laboratory measurements were those closest to randomization (preceding 4 days or 1 day post-randomization); for vital signs, the highest value within 24 hours of randomization was taken. Abbreviation: NA, not available.

^a^Not available due to participant deidentification.

^b^People with Child-Pugh C liver cirrhosis were excluded from the SAFO trial.

^c^Peripheral vascular disease, myocardial infarction, or stroke.

^d^Implanted cardiac devices, including pacemakers, implantable automatic cardioverter-defibrillator, and left ventricular assist devices, but not including prosthetic heart valves.

^e^Vertebral osteomyelitis, epidural abscess, native joint septic arthritis, prosthetic joint infection, deep tissue abscess.

^f^Injection drug use and bone classified as “other.”

^g^“Other” sources not specified.

^h^Data shown for trial control arms.

### Identification of Subphenotypes Using LCA

Eighteen class-defining variables were included in the final LCA. Despite colinearity, both creatinine and chronic kidney disease were included because creatinine provides additional information on the presence of acute kidney injury (correlation coefficient, 0.66). After determination of contributing variables (Supplementary Figure 2) using the Edinburgh cohort, latent class models with 1 to 7 classes were fitted (Table 2). For the Edinburgh and ARREST cohorts, the BIC and clinical interpretability favored the 5-class model, and the size of the smallest class was acceptable (>5% of the total population).

**Table 2.** Model Fit Statistics

Model	Bayesian Information Criteria^a^	Log-Likelihood^b^	Number of Parameters^c^	Entropy^d^	Patients per Class						
Edinburgh cohort											
					1	2	3	4	5	6	7
1-class	14 578.4	−7200.3	29	1	458	…	…	…	…	…	…
2-class	14 167.1	−6902.8	59	0.8	307	151	…	…	…	…	…
3-class	13 968.5	−6711.6	89	0.8	173	152	133	…	…	…	…
4-class	13 901.7	−6586.3	119	0.8	162	128	127	41	…	…	…
5-class	13 829.1	−6458.1	149	0.9	147	132	103	39	37	…	…
6-class	13 869.1	−6383.2	149	0.9	130	123	83	42	42	38	…
7-class	13 980.5	−6350.0	209	0.9	113	103	65	57	42	41	37
ARREST cohort											
					1	2	3	4	5	6	7
1-class	19 279.6	−10 157.3	26	1	758	…	…	…	…	…	…
2-class	18 636.4	−9741.5	53	0.8	576	182	…	…	…	…	…
3-class	18 360.2	−9515.8	80	0.8	410	232	116	…	…	…	…
4-class	18 284.9	−9374.1	107	0.8	284	197	162	115	…	…	–
5-class	18 269.8	−9259.3	134	0.8	276	139	121	115	107	…	…
6-class	19 409.7	−9171.1	161	0.8	171	153	119	116	106	93	…
7-class	19 462.4	−9107.9	188	0.8	172	131	117	112	88	78	60
SAFO cohort											
					1	2	3	4	5	6	7
1-class	2065.0	−976.1	21	1	214	…	…	…	…	…	…
2-class	1963.3	−866.3	43	0.8	125	89	…	…	…	…	…
3-class	1945.9	−798.6	65	0.9	125	67	22	…	…	…	…
4-class	1981.6	−757.4	87	0.8	79	71	44	20	…	…	…
5-class	2035.2	−725.1	109	0.9	59	54	45	36	20	…	…
6-class	2121.1	−709.1	131	0.9	58	53	38	34	18	13	…
7-class	2174.1	−676.5	153	0.9	51	40	36	37	23	20	7

Model fit statistics for latent class models from 1 to 7 classes in the Edinburgh, ARREST, and SAFO cohorts.

^a^Defined in Methods section.

^b^Measures the fit of the model to the data.

^c^Measure of model complexity.

^d^Measure for class separation; it ranges from zero to 1; values ≥0.8 indicate good separation of the different classes.

**Figure 1.**
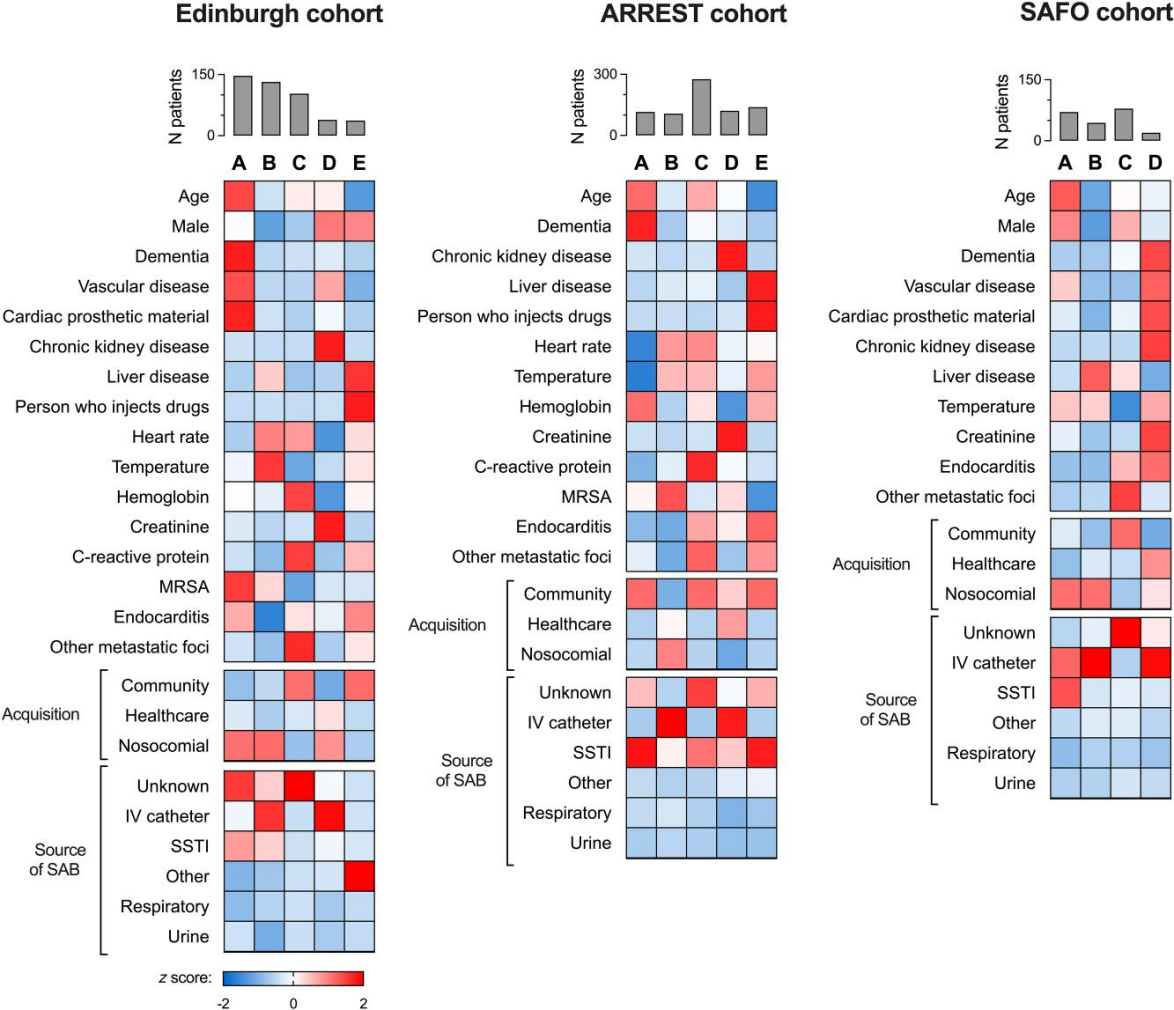
Comparison of class-defining variables for SAB subphenotypes. Vertical bars show the number of patients assigned to each subphenotype. Cells are shaded according to row *z* score (ie, comparing subphenotypes) except “Acquisition” and “Source of SAB,” where shading is by column *z* score (ie, comparing within each subphenotype). Intensity of red shading reflects a more positive *z* score (ie, above the mean), and intensity of blue shading reflects a more negative *z* score (ie, below the mean). In the SAFO trial, people with Child-Pugh C liver cirrhosis or MRSA infection and people who inject drugs were not recruited. Abbreviations: IV, intravenous; MRSA, methicillin-resistant *Staphylococcus aureus*; SAB, *Staphylococcus aureus* bacteremia; SSTI, skin or soft tissue infection.

The 5 classes identified by LCA in the Edinburgh and ARREST cohorts represented distinct clinical subphenotypes when their association with class-defining clinical variables was considered and were replicated in the 2 analyses (Figure 1). Subphenotype A was associated with older age, comorbidity, and SAB from an unknown or SSTI source. Subphenotype B was associated with nosocomial SAB, bacteremia originating from an intravenous catheter, younger age, less comorbidity, and lack of any metastatic foci. Subphenotype C was associated with community-acquired SAB from an unknown source, with higher C-reactive protein (CRP) and with the presence of metastatic foci of infection. Subphenotype D was associated with chronic kidney disease (CKD), intravenous catheter source, and nosocomial or healthcare-associated acquisition. In the Edinburgh cohort, 17 of 39 predicted members of this subphenotype received hemodialysis. Subphenotype E was associated with community-acquired SAB, younger age, IDU, liver disease, and endocarditis. In the Edinburgh cohort, the source of SAB in 32 of 37 predicted members of this subphenotype was IDU (categorized as “other” source in the LCA since this category did not exist in the classification used in ARREST). In the ARREST cohort, SSTI was the source enriched in subphenotype E, which is consistent with acquisition through IDU. In the Edinburgh cohort, 14 of 37 predicted members of this subphenotype had infected deep vein thrombophlebitis and 3 of 37 had an infected pseudoaneurysm.

The Infectious Diseases Society of America [26] and Fowler et al [27] definitions of complicated SAB were applied to the Edinburgh cohort, identifying a lower proportion of patients who met both definitions of complicated SAB in subphenotype B (nosocomial intravenous catheter SAB) and a higher proportion in subphenotype C (community-acquired metastatic SAB; Supplementary Figure 3A). Two current definitions of “low-risk” SAB were also applied to the Edinburgh cohort (the SABATO trial eligibility criteria [28, 29] and the definition used by Hendriks et al [30]), with patients meeting these definitions predominantly predicted to belong to subphenotype B (Supplementary Figures 3B and 3C). The distribution of *spa* type inferred clonal complexes (Supplementary Figure 3D) did not differ substantially between subphenotypes in the Edinburgh cohort. Predicted members of subphenotypes A (older comorbid SAB) and D (CKD SAB) in the Edinburgh cohort had the highest Charlson comorbidity index, with the predicted members of subphenotypes B and E having the lowest (Supplementary Figure 3E).

The SAFO trial included a substantially smaller number of participants than the Edinburgh and ARREST cohorts, applied more stringent inclusion and exclusion criteria (excluding MRSA infection, moderate to severe heart failure, and IDU), and did not record baseline CRP. Combining model fit parameters and interpretability, a 4-class model was favored. As expected, subphenotype E (IDU SAB) was not identified, but the other 4 classes identified were similar to subphenotypes A–D identified in the Edinburgh and ARREST cohorts (Figure 1). SAFO subphenotype A was associated with older age, vascular disease, and SAB originating from SSTI. SAFO subphenotype B was associated with younger patients with nosocomial SAB from an intravenous catheter source. SAFO subphenotype C was associated with community-acquired metastatic SAB from an unknown source. SAFO subphenotype D was associated with healthcare-associated SAB, chronic kidney disease, and an intravenous catheter source. Predicted members of SAFO subphenotypes A and D had the highest Charlson comorbidity index, and subphenotype B had the lowest, consistent with the associations seen in the Edinburgh cohort (Supplementary Figure 4).

### Clinical Outcomes of SAB Subphenotypes

**Figure 2.**
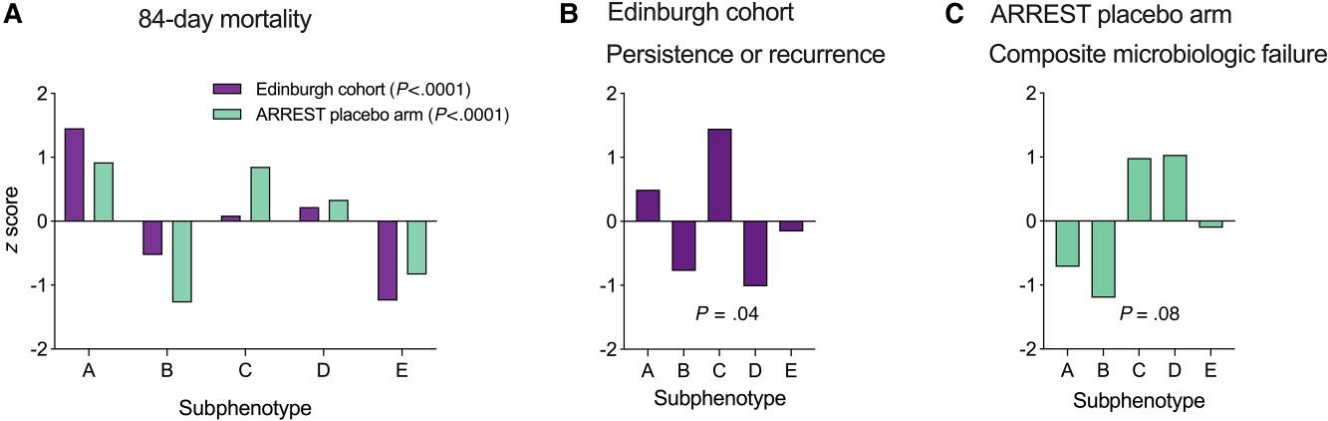
Comparison of outcomes between *Staphylococcus aureus* bacteremia (SAB) subphenotypes. Comparison of all-cause 84-day mortality (*A*), persistent or recurrent bacteremia in the Edinburgh retrospective observational cohort (*B*), and composite microbiologic failure in the ARREST trial placebo arm (*C*). Bars represent *z* scores comparing the outcome between subphenotypes within the same cohort. Differences in the proportion of patients with each outcome between subphenotypes were compared using the Fisher exact test or *χ*^2^ test.

Differences in 84-day mortality and microbiologic outcomes were observed between the subphenotypes (Figure 2*A*, Supplementary Table 1). In both the Edinburgh cohort and ARREST placebo arm (n = 388), 84-day mortality was highest in subphenotype A and lowest in subphenotypes E and B. In the Edinburgh cohort, people assigned to subphenotypes A and D had the lowest 1-year survival, whereas those assigned to subphenotype E had the highest (Supplementary Figure 5). In the Edinburgh cohort, subphenotype C was associated with increased rates of persistent or recurrent bacteremia (Figure 2*B*). In the ARREST placebo arm cohort, subphenotype C was also associated with a higher rate of composite microbiologic failure (Figure 2*C*). In both cohorts, subphenotype B was associated with lower rates of microbiologic failure. The smaller number of patients in the SAFO control arm (n = 110) limited our ability to compare outcomes between the subphenotypes, but similar patterns were observed (Supplementary Figure 6). People assigned to subphenotypes A and D had higher 84-day mortality. Patients assigned to subphenotype B had the lowest mortality and lowest rate of persistent bacteremia. Persistent bacteremia at day 7 was uncommon but mostly occurred in subphenotype C.

### Secondary Analysis of the Effect of Adjunctive Rifampicin Treatment Stratified by SAB Subphenotype

**Figure 3.**
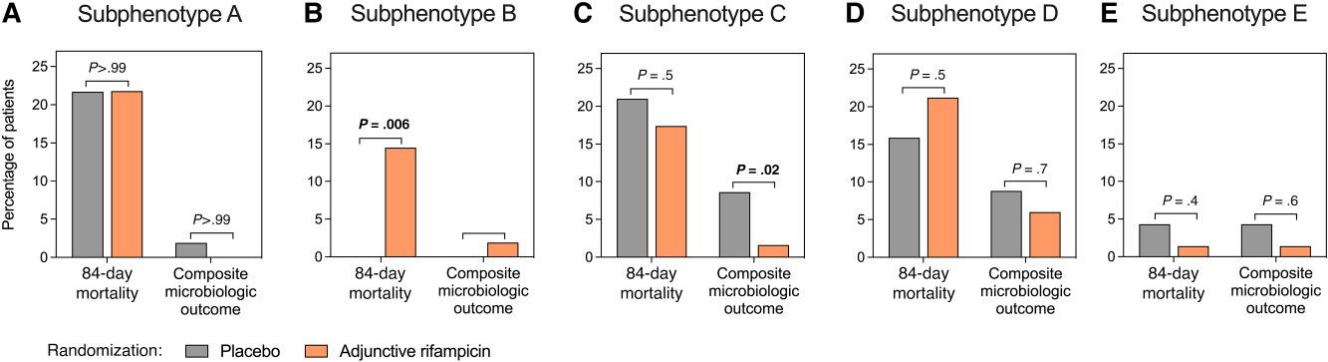
Effect of adjunctive rifampicin in *Staphylococcus aureus* bacteremia (SAB) subphenotypes. Comparison of outcomes of patients randomized to placebo or adjunctive rifampicin when SAB subphenotypes were considered separately. Treatment outcomes within each subphenotype were compared using the Fisher exact test. Because 2 comparisons were made within each subphenotype, the significance level was set at 0.025 (α=0.05, n = 2).

Application of stratification of patients with SAB into subphenotypes is to enrich clinical trial design. Within the ARREST cohort, we considered each subphenotype separately and within each compared the effect of adjunctive rifampicin on 84-day mortality and composite microbiologic failure (Figure 3, Supplementary Table 2). Patients assigned to subphenotype B and randomized to adjunctive rifampicin had a higher 84-day mortality rate compared with patients randomized to placebo (odds ratio [OR], 18.8; 95% confidence interval [CI], 1.1–334.4; *P* = .006). In subphenotype C, randomization to adjunctive rifampicin was associated with reduced composite microbiologic failure (OR, 0.17; 95% CI, .04–.8; *P* = .02).

## DISCUSSION

In hospitalized patients with predominantly MSSA bacteremia, 5 subphenotypes can be identified using routinely available clinical data. These subphenotypes differ in survival and microbiologic outcomes. In a hypothesis-generating secondary analysis of the ARREST trial, differential treatment effects were observed. Adjunctive rifampicin was associated with increased 84-day mortality in 1 subphenotype (nosocomial intravenous catheter SAB) and an improved microbiologic outcome in another (community-acquired metastatic SAB).

Our findings permit several observations about SAB from an unbiased standpoint. Subphenotype B (nosocomial intravenous catheter SAB) represents patients at low risk of adverse outcomes. A total of 132 patients (28.8%) were predicted to belong to this subphenotype in the Edinburgh cohort, whereas 71 (15.5%) met the inclusion criteria for the SABATO trial [28, 29] and 83 (18.1%) met the Hendriks et al definition of low-risk SAB [30], with the majority predicted to belong to subphenotype B. Subphenotype B could therefore represent a rational target for expanded investigation of earlier oral switch in SAB, providing a data-driven definition of low-risk SAB. Furthermore, the risks of adjunctive agents might outweigh the limited potential to improve on already good outcomes, as exemplified by our finding that adjunctive rifampicin potentially caused increased mortality in this subphenotype. Inclusion of this subphenotype in trials of combination therapy should be done cautiously. Subphenotype E (IDU SAB) was associated with complicated disease and, despite this, low mortality. Subphenotype C (community-acquired metastatic SAB) had complicated disease and worse microbiologic outcomes but without clear predisposing factors. Patients in this subphenotype had a lower Charlson comorbidity index and generally lacked an obvious source for bacteremia. The possible benefit of adjunctive rifampicin warrants further investigation in this subphenotype, in addition to alternative adjunctive agents including antimicrobials (eg, clindamycin, currently being evaluated in the adjunctive treatment domain of the SNAP trial [31]) and anti-staphylococcal lysins (Exebacase) [32]. These subphenotypes also provide a framework for investigation of immunobiology in SAB and could facilitate identification of treatable traits, for example, defective phagocyte responses that could be therapeutically recalibrated [11].

Our study has several strengths. The subphenotypes were replicated in analysis of an observational cohort and a large trial cohort with permissive inclusion criteria. Four of the subphenotypes could also be identified in a smaller trial with more restrictive inclusion criteria. Trial populations of SAB differ from real-life cohorts, including patient characteristics and mortality rates [9, 29]. It is therefore reassuring that despite the differences between the cohorts (Table 1), the core features of the identified subphenotypes were reproducible, suggesting that the findings are generalizable. Outcomes differed between subphenotypes but were not included as class-defining variables and, overall, the association between subphenotype and outcome was consistent across the cohorts. To allow prospective subphenotype prediction of individual patients, future work will aim to identify a subset of variables that can be used as predictive markers of subphenotype membership.

Our study has important limitations. First, despite using model parameters such as BIC, there is a degree of subjectivity with the class selection based on clinical interpretability. Second, the class-defining variables included were restricted to routinely available clinical data. Inclusion of inflammation biomarkers could provide biological insights. Third, the included cohorts were from countries with a low prevalence of MRSA. The USA300 MRSA clone is prevalent in the United States and independently associated with metastatic disease [3]. Replication in a cohort with higher MRSA prevalence will be required. Fourth, the cohorts differed in inclusion criteria and the variables available for analysis. Fifth, the definitions of microbiologic outcomes used in the cohorts were different, preventing direct comparison. Sixth, the Edinburgh cohort was a retrospective observational study without structured prospective monitoring of microbiologic outcomes. Detection of persistent or recurrent SAB was opportunistic, relying on healthcare attendance and blood cultures being taken, and so is likely to be subject to ascertainment bias and underascertainment of these outcomes. Finally, although receipt of adjunctive rifampicin was randomized in the ARREST trial, reducing the risk of confounding, the numbers within each subphenotype were relatively small. Consequently, these results must be interpreted as strictly hypothesis-generating. Overall, it remains possible that the subphenotypes will not be replicable in other patient cohorts, that additional subphenotypes may exist (eg, those associated with MRSA infection), or that outcomes/treatment responses could differ. We are conducting further replication studies to address these questions.

In summary, our findings support the hypothesis that clinically relevant subphenotypes do exist within SAB and suggest that patient stratification within SAB clinical trials is required to identify strategies to improve outcomes for patients. This could inform a personalized medicine approach to SAB.

## Supplementary Data

Supplementary materials are available at *Clinical Infectious Diseases* online. Consisting of data provided by the authors to benefit the reader, the posted materials are not copyedited and are the sole responsibility of the authors, so questions or comments should be addressed to the corresponding author.

## Supplementary Material

ciae338_Supplementary_Data

## Notes

***Author Contributions***. C. D. R. conceived the study. C. D. R., G. E. T., M. P., J. N. P., C. T., J. C., A. S., K. B., G. C., W. S., R. S. L., T. Y. G., W. D., A. B., E. H., and L. T. curated the data. M. C. S., Z. B., A. C. W., G. H. G., and C. D. R. performed the investigation. M. C. S., Z. B., and C. D. R. performed the formal analysis. C. D. R., M. C. S., and G. H. G. supervised the study. M. C. S. and C. D. R. wrote the original draft of the manuscript. All authors reviewed and edited the manuscript. C. D. R. and M. C. S. validated the study data. M. C. S., C. D. R., A. S., G. E. T., M. P., J. N. P., C. T., and J. C. had access to the raw data. The corresponding author had full access to all of the data and final responsibility for the decision to submit for publication.

***Acknowledgments***. This work was based on data provided by patients and collected by the National Health Service (NHS) as part of their care. The authors gratefully acknowledge the work of the ARREST and SAFO trial teams (listed in the Supplementary Material).

***Disclaimer.*** The views expressed in this publication are those of the authors and not necessarily those of the NHS, the National Institute for Health Research, or the Department of Health.

***Financial support***. ARREST was funded by the National Institutes of Health Research's Health Technology Assessment (project 10/104/2). The Medical Research Council (MRC) Clinical Trials Unit at University College London is supported by funding from the MRC (MC_UU_00004/05). M. P. received a research grant from Laboratorios ERN. V. G. F. was supported by grant R01-AI165671 from the National Institutes of Health. This work was supported by antimicrobial resistance cross-council funding from the MRC to the SHIELD consortium “Optimising Innate Host Defence to Combat Antimicrobial Resistance” (PI: D. H. D.). C. D. R. was supported by an Edinburgh Clinical Academic Track (ECAT)/Wellcome Trust PhD Training Fellowship for Clinicians award (214178/Z/18/Z). This research was funded in part by Wellcome 214178/Z/18/Z. For the purpose of open access, the author has applied a Creative Commons attribution (CC-BY) license to any author-accepted manuscript version arising.

***Data sharing***. Please contact the corresponding author to discuss access to the dataset. We welcome opportunities to share data and contribute to collaborative analyses.
