# Supplementary material for: Clinical Subphenotypes of Staphylococcus aureus Bacteremia
Source: Clin Infect Dis. 2024 Jun 25;79(5):1153–61. doi: 10.1093/cid/ciae338 (PMC11581694; doi:10.1093/cid/ciae338)
Supplement: ciae338_Supplementary_Data [file ciae338_supplementary_data.pdf]

Supplementary material

Contents:

Supplementary Table 1: Outcomes in Edinburgh and ARREST cohorts p2
Supplementary Table 2: Effect of adjunctive rifampicin stratified by sub-phenotype p3
Supplementary Figure 1: Flow diagram for Edinburgh retrospective observational cohort p4
Supplementary Figure 2: Selection of class defining variables p5
Supplementary Figure 3: Additional features of sub-phenotypes in the Edinburgh cohort p6
Supplementary Figure 4: Median Charlson Comorbidity Index of sub-phenotypes in the SAFO cohort p7
Supplementary Figure 5: One-year survival of sub-phenotypes in the Edinburgh cohort p8
Supplementary Figure 6: Outcomes in the SAFO cohort p9
List of ARREST investigators p10
List of SAFO investigators p12

Supplementary Table 1: Outcomes in Edinburgh and ARREST cohorts

	Sub-phenotype					P-value
	A	B	C	D	E	
**Edinburgh cohort**	n=147	n=132	n=103	n=39	n=37	
84-day mortality	64 (43·8)	20 (15·5)	25 (24·3)	10 (26·2)	2 (5·3)	<0·0001
Persistence or recurrence	7 (4·8)	1 (0·8)	8 (7·8)	0	1 (2·7)	0·04
**ARREST placebo arm**	n=60	n=52	n=138	n=69	n=69	
84-day mortality	13 (21·7)	0	29 (21·0)	11 (15·9)	3 (4·3)	<0·0001
Composite microbiologic failure	1 (1·9)	0	11 (8·6)	6 (8·8)	3 (4·3)	0·08

Results show the percentage of patients in each sub-phenotype with the specific outcome. Data shown as n (%). Outcomes were compared between sub-phenotypes within each cohort using Fisher's exact test.

Supplementary Table 2: Effect of adjunctive rifampicin stratified by sub-phenotype

	Placebo	Adjunctive rifampicin	P-value
**Sub-phenotype A**	n=60	n=55	
84-day mortality	13 (21·7)	12 (21·8)	>0·99
Composite microbiologic failure	1 (1·9)	0 (0)	>0·99
**Sub-phenotype B**	n=52	n=55	
84-day mortality	0 (0)	8 (14·5)	0·006
Composite microbiologic failure	0 (0)	1 (1·9)	>0·99
**Sub-phenotype C**	n=138	n=138	
84-day mortality	29 (21·0)	24 (17·4)	0·54
Composite microbiologic failure	11 (8·6)	2 (1·6)	0·02
**Sub-phenotype D**	n=69	n=52	
84-day mortality	11 (15·9)	11 (21·2)	0·48
Composite microbiologic failure	6 (8·8)	3 (6·0)	0·73
**Sub-phenotype E**	n=69	n=70	
84-day mortality	3 (4·3)	1 (1·4)	0·37
Composite microbiologic failure	3 (4·3)	1 (1·4)	0·62

Treatment outcomes within each sub-phenotype were compared using Fisher's exact test. Data shown as n (%). Two comparisons were made within each sub-phenotype so the significance level was set at 0·025 (α=0·05, n=2).

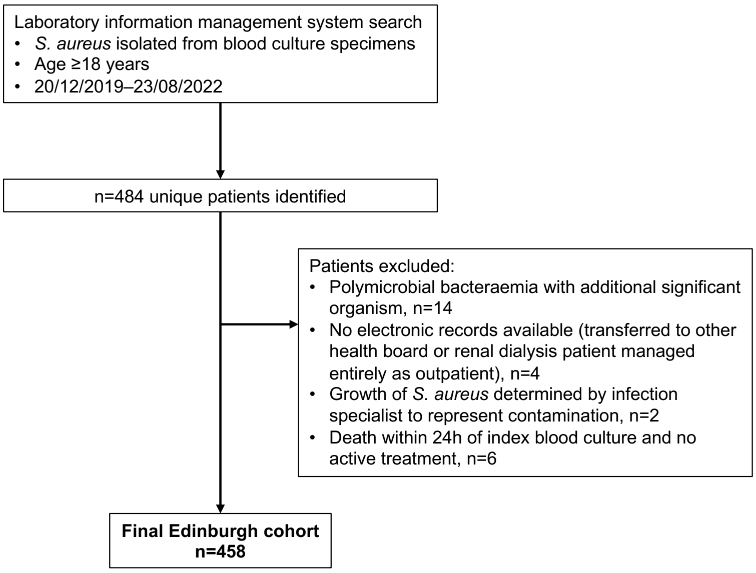

Supplementary Figure 1: Flow diagram for Edinburgh retrospective observational cohort

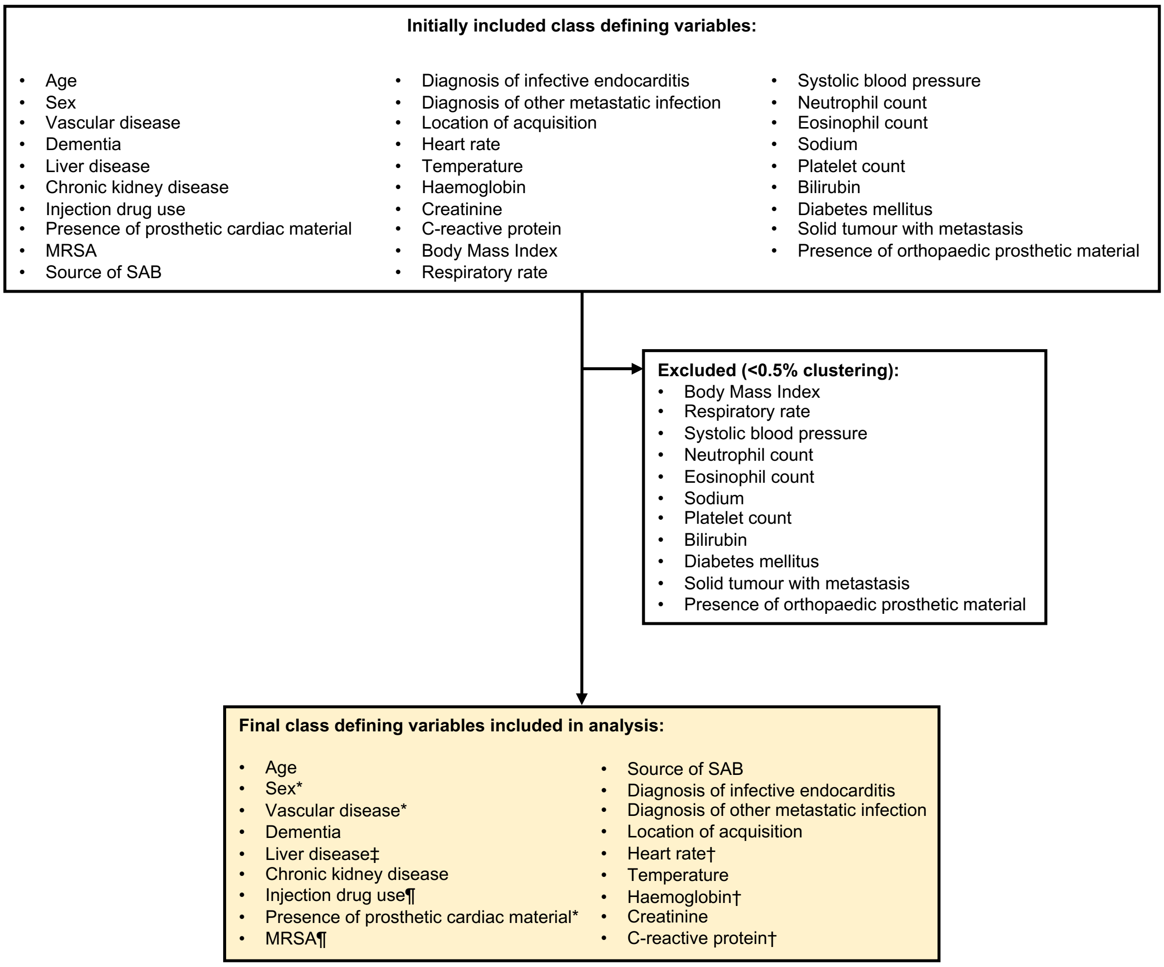

Supplementary Figure 2: Selection of class defining variables

Exclusions were based on contribution to clustering within the Edinburgh cohort. Variables marked with * were not available for the ARREST cohort and † were not available for the SAFO cohort.

¶ people who inject drugs and people with MRSA bacteraemia were excluded from the SAFO trial.

‡ people with Child-Pugh C liver cirrhosis were excluded from the SAFO trial.

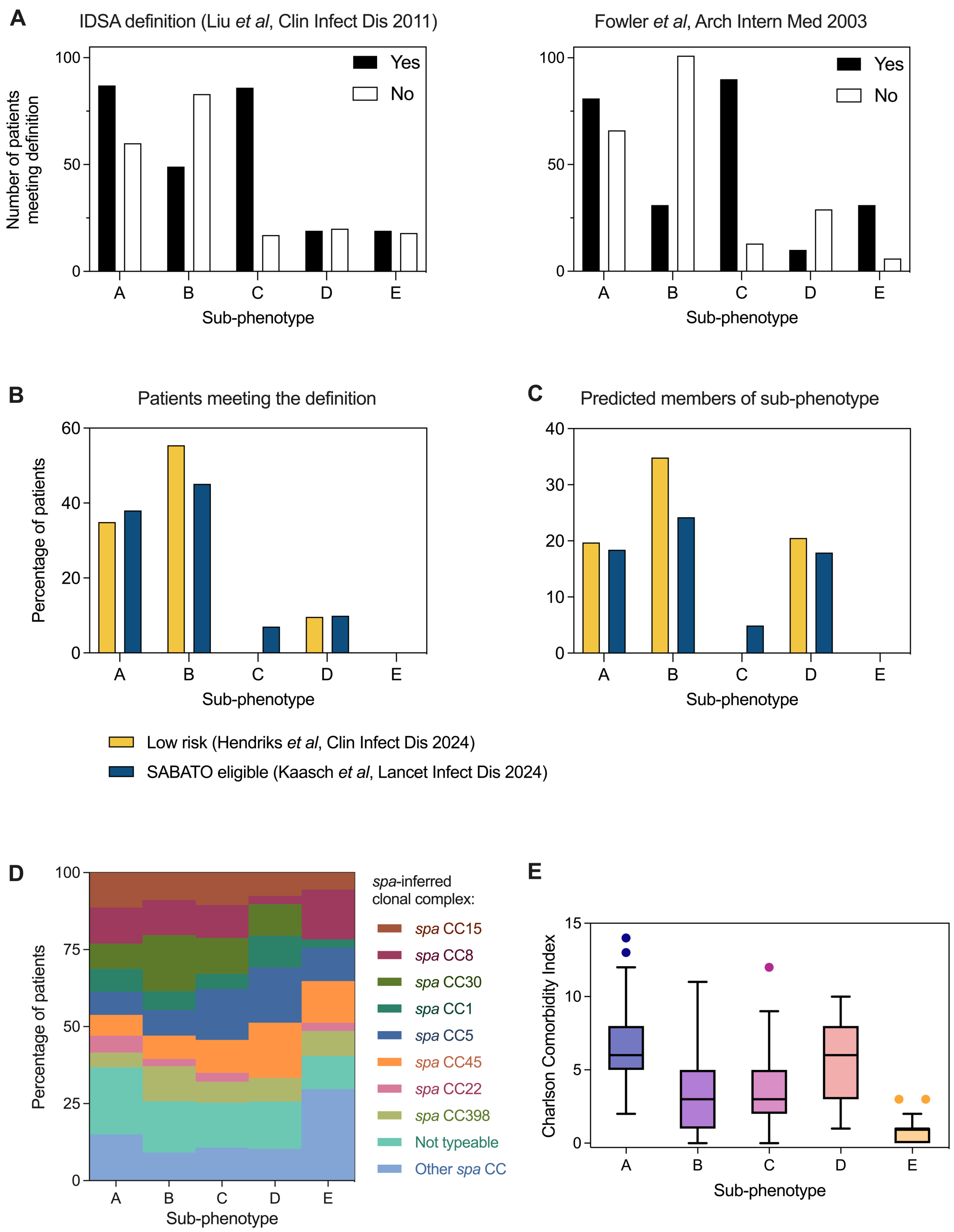

Supplementary Figure 3: Additional features of sub-phenotypes in the Edinburgh cohort
(A) Proportion of patients in the Edinburgh cohort predicted to belong to each sub-phenotype, stratified by presence of complicated SAB using two different definitions. **(B)** Proportion of patients meeting two different definitions of low-risk SAB, stratified by predicted sub-phenotype membership (*i.e.* denominator is total number of patients meeting the definition). **(C)** Proportion of patients predicted to belong to each sub-phenotype meeting the definitions of low-risk SAB (*i.e.* denominator is total number of patients predicted to belong to each sub-phenotype. **(D)** *spa*-inferred clonal complex of *S. aureus* bloodstream isolates from the Edinburgh cohort, stratified by sub-phenotype. **(E)** Charlson Comorbidity Index of patients in the Edinburgh cohort, stratified by sub-phenotype. Box and whisker plot drawn using Tukey's method. Box shows interquartile range and horizontal line shows median.

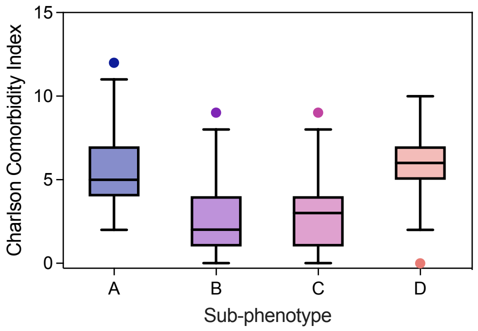

Supplementary Figure 4: Median Charlson Comorbidity Index of sub-phenotypes in the SAFO cohort
Charlson Comorbidity Index of patients in the SAFO cohort, stratified by sub-phenotype. Box and whisker plot drawn using Tukey's method. Box shows interquartile range and horizontal line shows median.

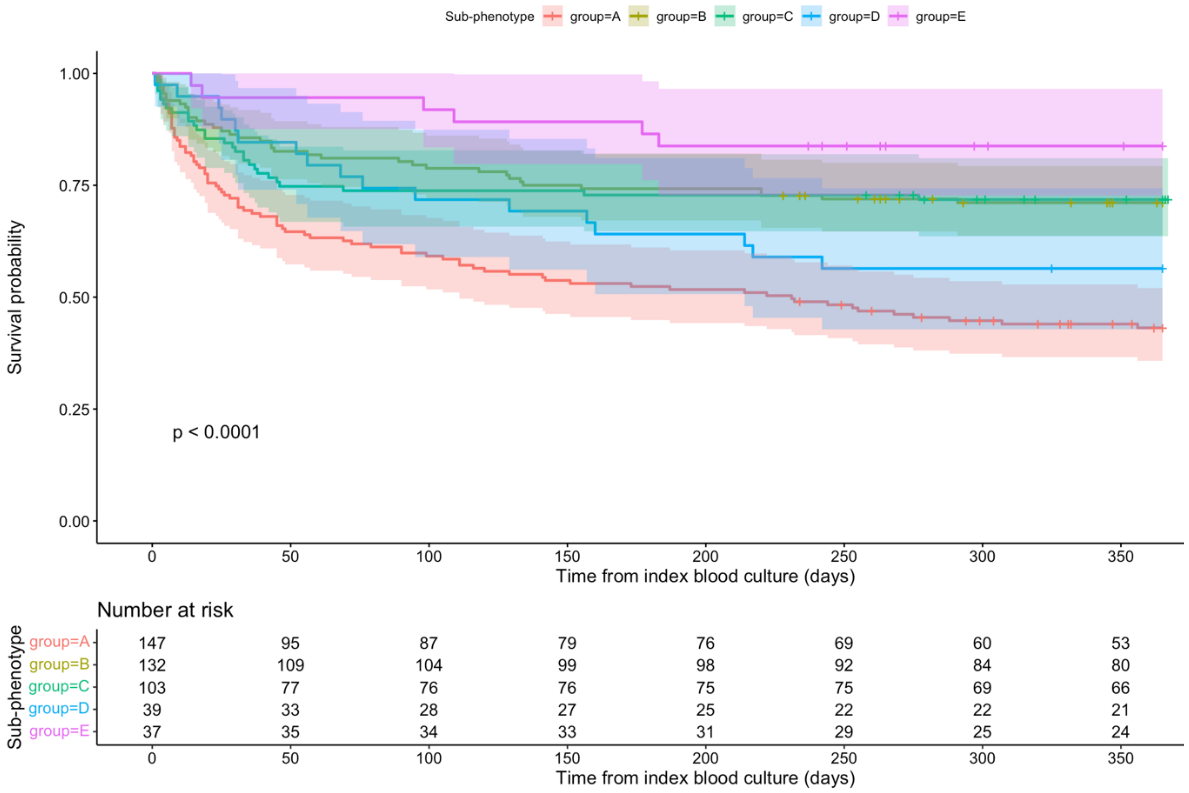

Supplementary Figure 5: One-year survival of sub-phenotypes in the Edinburgh cohort

Unadjusted 365-day survival. Kaplan-Meier survival curve, shaded area represents 95% confidence interval for percentage surviving. Sub-phenotypes compared using the log-rank test. Group designations correspond to sub-phenotypes.

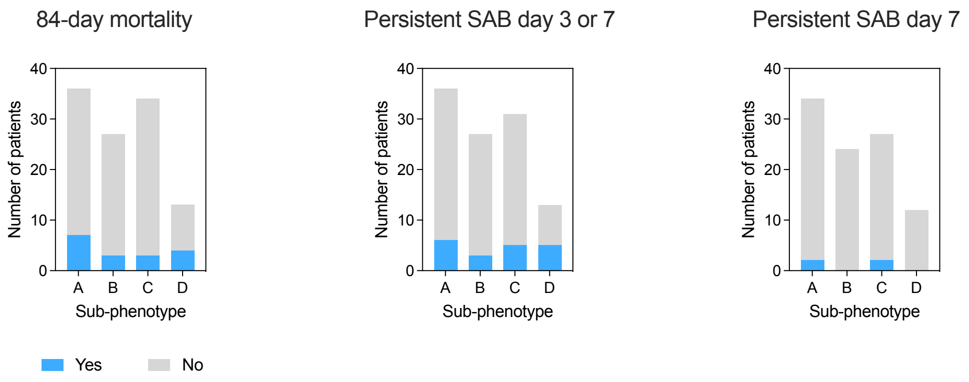

Supplementary Figure 6: Outcomes in the SAFO cohort
Data shown for the SAFO trial control arm participants.

List of ARREST investigators

The ARREST trial group consists of:

Participating Centres:
Oxford University Hospitals NHS Trust: M Scarborough, M Kamfose, A de Veciana, NC Gordon, L Peto, G Pill, T Clarke, L Watson, B Young, D Griffiths, A Vaughn, L Anson, E Liu, S Perera, L Rylance-Knight, C Cantell, R Moroney.
Guy's and St Thomas' NHS Foundation Trust: JD Edgeworth, G Thwaites, K Bisnauthsing, A Querol-Rubiera, C Gibbs, A Patel, C Hemsley, AL Goodman, D Wyncoll, J Biswas, J Fitzpatrick, L Roberts, J Millard, N Stone, A Cape, L Hurley, C Kai Tam.
The Royal Liverpool and Broadgreen University Hospitals NHS Trust: E Nsutebu, M Hoyle, K Maitland, L Trainor, H Reynolds, J Harrison, J Anson, J Lewis, J Folb, L Goodwin, N Beeching, S Dyas, H Winslow, E Foote, P Roberts, P Natarajan, A Chrdle. M Fenech, H Allsop.
Plymouth Hospitals NHS Trust: R Tilley, R Austin-Hutchison, L Barrett, K Brookes, L Carwithen, A Conbeer, R Cunningham, C Eglinton, R Fok, H Gott, S Hughes, L Jones, M Kalita, A King, L March, M Marner, T Mynes, A Plant, S Price, J Sercombe, A Stolton, M Wallis, M West, J Westcott, C Williams, R Wosley, L Yabsley.
Sheffield Teaching Hospitals NHS Foundation Trust: J Greig, L Butland, J Sorrell, T Mitchell, A Alli, J Meiring, B Masake, C Rowson, L Smart, L Makey, S Moll, J Cunningham, K Ryalls, K Burchall, J Middle, Y Jackson, D Swift, J Cole, B Subramanian, F Okhuoya, M Edwards, C Bailey, R Warren, G Islam, M Ankcorn, S Birchall, P Jones, J Humphries, S Booth, C Evans.
Portsmouth Hospitals NHS Trust: S Wyllie, A Flatt, L Strakova, M Hayes, S Valentine, C James, M Wands, N Cortes, N Khan, R Porter, Z Martin, K Yip, H Preedy, H Chesterfield, T Dobson, C Walker.
Brighton and Sussex University Hospitals NHS Trust: M Llewelyn, A Dunne, L Latter, A Porges, J Price, J Paul, L Behar, L Robinson, A Murray, J Fitzpatrick, T Sargent, C Ridley, L Ortiz-Ruiz de Gordoa, D Gilliam, C McPherson, S Matthews, E Foreman, R Jarghese, A Beddoe, S Martin, S Shaw, D Wlazly, M Cole, A Gihawi, K Cole.
Cambridge University Hospitals NHS Foundation Trust: ME Török, T Gouliouris, L Bedford, RB Saunderson, I Mariolis, R Bousfield, I Ramsay, D Greaves, S Aliyu, K Cox, L Mlemba, L Whitehead, N Vyse, M Bolton
South Tees Hospitals NHS Foundation Trust: J Williams, P Lambert, D Chadwick, K Baillie, M Cain, R Bellamy, J Wong, J Thompson, H Vassallo, A Skotnicka, A Boyce, A Donnelly.
University College London Hospitals NHS Foundation Trust: P Wilson, G FitzGerald, V Dean, K Warnes, A Reyes, S Rahman, L Tsang, J Williams, S Morris-Jones.
Royal Free London NHS Foundation Trust: S Hopkins, E Witness, O Brady, E Woodford, T Pettifer, A McCadden, B Marks, S Collier, D Mack, S Warren, C Brown, A Lyons, S Taiyari, S Mepham, A Sweeney, L Brown.
Royal Devon and Exeter NHS Foundation Trust: C Auckland, A Potter, J Mandiza, M Hough, S Williams, C Renton, F Walters, M Nadolski, M Hough, A Evans, P Tarrant, S Williams, K Curley, S Whiteley, J Halpin, M Hutchings, S Todd, C Lohan, T Chapter, E Folland, A Colville, K Marden, M Morgan, R Fok, R Porter, M Baxter.
The Leeds Teaching Hospital NHS Trust: J Minton, S Rippon, M Cevik, J Chapman, T Kemp, R Vincent, D Osborne, T Platt, J Calderwood, B Cook, C Bedford, L Galloway-Browne, N Abberley, K Attack, J Allen.
Aintree University Hospital NHS Foundation Trust: P Lal, M Harrison, S Stevenson, C Brooks, P Harlow, J Ewing, S Cooper, R Balancio-Tolentino, L O'Neil, R Tagney, D Shackcloth.
St George's Healthcare NHS Trust: T Planche, J Fellows, R Millett, J Studham, C de Souza, G Howell, H Greaves, E Foncel, R Kurup, J Briggs, M Smith, C Suarez, G Sorrentino, A Scobie, A Houston, F Ahmad, A Breathnach, R Chahuan, K Wilkins.
Blackpool Teaching Hospitals NHS Foundation Trust: A Guleri, N Waddington, R Sharma, P Flegg, V Kollipara, M Alam, A Potter, S Donaldson, C Armer, J Frudd.
King's College Hospital NHS Foundation Trust: D Jeyaratnam, M Joy, A Mathews, SK Glass, A Ajayi, A Fife, S Qaiser, S Sheehan, S Muñoz Villaverde, NO Yogo, I De Abreu, G Notcheva, J Flanagan, C Watson, E Sais, A Adedayo, V Chu, G Shaw, MA Graver, R Palmer, D Palmer, S Haile, J Gordon, C Kai Tam, Mandar K, W Szypura.
Heart of England NHS Foundation Trust: N Jenkins, J Marange, V Shabangu, K Moore, J Lyons, M Munang, M Sangombe, E Moran, A Hussain.

University Hospitals of Leicester NHS Trust: M Wiselka, A Lewszuk, S Batham, K Ellis, L Bahadur, H White, M Pareek, A Sahota, S Coleman, H Pateman, A Kotecha, C Sim, A Rosser.
County Durham and Darlington NHS Foundation Trust: D Nayar, J Deane, R Nendick, C Aldridge, A Clarke, M Wood, A Marshall, L Stephenson, T Matheson-Smith, J Sloss, K Potts, J Malkin, L Ftika, V Raviprakash.
University Hospital Southampton NHS Foundation Trust: J Sutton, A Malachira, M Kean, K Criste, K Gladas, C Andrews, C Hutchison, E Adams, J Andrews, B Romans, N Ridley, M Ekani, J Mitchell, N Smith, T Clark, S Glover, R Reed, T Yam, H Burton, R Said.
Wirral University Teaching Hospital NHS Foundation Trust: D Harvey, A Janvier, R Jacob, C Smalley, A Fair.
Dartford & Gravesham NHS Trust: A Gonzalez-Ruiz, S Lord, K Ripalda, H Wooldridge, L Cotter, G Cardoso, E Strachan, G Kaler, A Mohamoodally, E Lawrence, Z Prime, R Abrahams.
The Newcastle upon Tyne Hospitals NHS Foundation Trust: DA Price, L Rigden, L Shewan, K Cullen, I Emmerson, K Martin, H Wilson, C Higham, K L Taylor, E Ong, B Patel, H Bond, J Gradwell, J Widdrington.
North Cumbria University Hospitals: C Graham, S Thornthwaite, U Poultney, H Crowther, H Fairlamb, E Hetherington, C Brewer, S Banerjee, C Hamson, A McSkeane.
Bradford Teaching Hospitals NHS Foundation Trust: P McWhinney, P Sharratt, J Thorpe, S Kimachia, H Wilson B Jeffs, L Masters, J Wilson, J Platt, L Burgess.
Salford Royal NHS Foundation Trust: P Chadwick, A Jeans, C Keatley, A Moran, Z Swann, K Pagett, A Peel, J Howard.
Royal United Hospital Bath NHS Trust: S Meisner, K Maloney, A Masdin, L Wright.
Hull and East Yorkshire Hospitals NHS Trust: G Barlow, S Crossman, V Lowthorpe, E Moore, P Moss, A Parkin, A Wolstencroft, B Warner, C Tarbotton, A Eyre, A Anderson, T Burdett, A Driffill.

Trial Coordination and Oversight - MRC CTU at UCL, London: AS Walker, F Hudson, A Szubert, J Cairns, D Ward, H Webb, C Russell, B Jackson, D Otiko. **Data Management Systems**: C Borg, L Masters, Z Islam, C Diaz, D Johnson

Trial Steering Committee: (Independent) A Martineau (Chair), A Kaasch, G Scott, J Bostock. (Team) G Thwaites, AS Walker, G Barlow, S Hopkins.

Data Monitoring Committee: D Lalloo (Chair), M Wilcox, D Altman.

Independent Endpoint Review Committee: T Peto, G Cooke.

List of SAFO investigators

The SAFO trial group consists of:

Participating Centres:
Hospital Universitari de Bellvitge: S Grillo, M Pujol, D Berbel, S Videla, P Hereu, A Padullés, N Pallarès, C Tebé, MA Domínguez, RF Rigo-Bonnin, G Horna, D Mediavilla, M Sanllorente, J Carratalà.
Hospital Clinic de Barcelona: JM Miró, L Morata, G Cuervo, A Soriano, C Pitart.
Hospital de la Santa Creu i Sant Pau: J López-Contreras, A Izquierdo-Cárdenas, MA Rivera, V Pomar.
Hospital Universitario Cruces: G Euba, AJ Goikoetxea, M Aguirregabiria, R Rodríguez-Álvarez, MJ Blanco-Vidal.
Hospital Universitari Parc Taulí: O Gasch, A Gomila-Grange, M Alguacil-Guillen,
Hospital Universitari Mútua Terrassa: L Boix-Palop, B Dietl, M Xercavins.
Hospital Universitario Lucus Agusti: MJ García-País.
Complexo Hospitalario Universitario de Vigo: MT Pérez-Rodríguez, A Sousa, FJ Vasallo-Vidal.
Hospital del Mar: S Gómez-Zorrilla.
Hospital de Sant Joan Despi Moises Broggi: I Oriol, B Borjabad, A Coloma-Conde, R Clivillé-Abad, SX González-di Lauro.
Hospital Universitario Virgen Macarena: LE López-Cortés, M de Cueto, E Moreno-Mellado.
Hospital Universitari Germans Trias i Pujol: ML Pedro-Botet.
Hospital 12 de Octubre: R San-Juan, JM Aguado, J Tiago-Silva, MA Orellana.
Hospital Universitario Ramón y Cajal: F Gioia, P Vizcarra, AM Sánchez-Díaz.
Hospital Universitari de Sant Joan: S Iftimie, C Garcia, F Ballester.
Hospital Universitari Arnau de Vilanova: A Jover-Sáenz, MF Ramírez-Hidalgo, A Bellés-Bellés.
Hospital Universitari de Tarragona Joan XXIII: G García-Pardo, E Picó-Plana.
Hospital Universitario La Paz: B Loeches, M Ruíz-Bastian.
Laboratori de Referència de Catalunya-UPF: A Siverio-Parés.
Hospital de Barcelona-SCIAS: Y Meije, A Ribera, J Llaberia.

Trial Coordination and Oversight: S Grillo, M Pujol, J Carratalà. **Data Management Systems**: N Pallarès, C Tebé.

Trial Steering Committee: M Pujol (Chair), JM Miró, S Videla, J Carratalà.

Data Monitoring Committee: P Hereu (Chair), G Horna, D Mediavilla, M Sanllorente.

Independent Endpoint Review Committee: D Chivite, D Rodríguez, C Tebé.
